# Peptidomic analysis of the venom of the solitary bee *Xylocopa appendiculata circumvolans*

**DOI:** 10.1186/s40409-017-0130-y

**Published:** 2017-08-29

**Authors:** Kohei Kazuma, Kenji Ando, Ken-ichi Nihei, Xiaoyu Wang, Marisa Rangel, Marcia Regina Franzolin, Kanami Mori-Yasumoto, Setsuko Sekita, Makoto Kadowaki, Motoyoshi Satake, Katsuhiro Konno

**Affiliations:** 1 Institute of Natural Medicine, University of Toyama, 2630 Sugitani, Toyama, 930-0194 Japan; 2 Faculty of Agriculture, Utsunomiya University, Utsunomiya, Tochigi Japan; 3 Immunopathology Laboratory, Butantan Institute, Sao Paulo, SP Brazil; 4 Department of Physiological Sciences, Institute of Biological Sciences, University of Brasília, Brasília, Brazil; 5 Bacteriology Laboratory, Butantan Institute, Sao Paulo, SP Brazil; 6 Faculty of Pharmaceutical Sciences at Kagawa Campus, Tokushima Bunri University, Sanuki, Kagawa Japan; 7 Laboratory of Plant Resources for Medicine, Showa Pharmaceutical University, Machidashi, Tokyo Japan

**Keywords:** Peptidomic analysis, LC-ESI-MS, Solitary bee, Venom, Linear cationic α-helical peptide

## Abstract

**Background:**

Among the hymenopteran insect venoms, those from social wasps and bees – such as honeybee, hornets and paper wasps – have been well documented. Their venoms are composed of a number of peptides and proteins and used for defending their nests and themselves from predators. In contrast, the venoms of solitary wasps and bees have not been the object of further research. In case of solitary bees, only major peptide components in a few venoms have been addressed. Therefore, the aim of the present study was to explore the peptide component profile of the venom from the solitary bee *Xylocopa appendiculata circumvolans* by peptidomic analysis with using LC-MS.

**Methods:**

A reverse-phase HPLC connected to ESI-OrbiTrap MS was used for LC-MS. On-line mass fingerprinting was made from TIC, and data-dependent tandem mass spectrometry gave MSMS spectra. A major peptide component was isolated by reverse-phase HPLC by conventional way, and its sequence was determined by Edman degradation, which was finally corroborated by solid phase synthesis. Using the synthetic specimen, biological activities (antimicrobial activity, mast cell devaluation, hemolysis, leishmanicidal activity) and pore formation in artificial lipid bilayer were evaluated.

**Results:**

On-line mass fingerprinting revealed that the crude venom contained 124 components. MS/MS analysis gave 75 full sequences of the peptide components. Most of these are related to the major and novel peptide, xylopin. Its sequence, GFVALLKKLPLILKHLH-NH_2_, has characteristic features of linear cationic α-helical peptides; rich in hydrophobic and basic amino acids with no disulfide bond, and accordingly, it can be predicted to adopt an amphipathic α-helix secondary structure. In biological evaluation, xylopin exhibited broad-spectrum antimicrobial activity, and moderate mast cell degranulation and leishmanicidal activities, but showed virtually no hemolytic activity. Additionally, the peptide was able to incorporate pores in artificial lipid bilayers of azolectin, confirming the mechanism of the cytolytic activity by pore formation in biological membranes.

**Conclusions:**

LC-ESI-MS and MS/MS analysis of the crude venom extract from a solitary bee *Xylocopa appendiculata circumvolans* revealed that the component profile of this venom mostly consisted of small peptides. The major peptide components, xylopin and xylopinin, were purified and characterized in a conventional manner. Their chemical and biological characteristics, belonging to linear cationic α-helical peptides, are similar to the known solitary bee venom peptides, melectin and osmin. Pore formation in artificial lipid bilayers was demonstrated for the first time with a solitary bee peptide.

## Background

Among the hymenopteran insects, the venoms from social wasps and bees – including honeybees, hornets and paper wasps – have been well documented [1, 2]. Their venoms are composed of a number of peptides and proteins and are used for defending their nests and themselves from predators. In contrast, venoms from solitary wasps and bees still require further research. In recent years, we have studied venoms from solitary wasps from Japan and found peptide neurotoxins, antimicrobial and cytolytic peptides and bradykinin-related peptides [3]. However, venoms from solitary bees have never been studied until quite recently.

The first study on solitary bee venoms was published only in 2008 about the European solitary bee *Melecta albifrons* [4]. A novel peptide, melectin, was isolated and characterized. Melectin has similar characteristics to those of melittin and mastoparan from the honeybee and hornet venoms. It is rich in hydrophobic and basic amino acids, amphipathic properties, and shows antimicrobial, mast cell degranulating and hemolytic activities. Accordingly, this peptide belongs to linear cationic α-helical peptides. Since then, studies describing similar solitary bee venom peptides have appeared: osmin [5], panurgine-1 [6], macropin [7], codesane [8], and HYL [9] (Table 1).

**Table 1:** Solitary bee venom peptides

Melectin	GFLSILKKVLPKVMAHMK-NH_2_
Osmin	GFLSALKKYLPIVLKHV-NH_2_
Panurgine-1	LNWGAILKHIIK-NH_2_
Macropin	GFGMALKLLKKVL-NH_2_
Codesane	GMASLLAKVLPHVVKLIK-NH_2_
HYL	GIMSSLMKKLAAHIAK-NH_2_
Xylopin	GFVALLKKLPLILKHLH-NH_2_
Xylopinin	GFVALLKKLPLILKHLP-NH_2_

These studies describe only the isolation and characterization of major peptides, which comprise a few components of the venom. However, such venoms consist of a complex mixture of many constituents, which cooperatively act for the venom toxicity and biological functionality. Accordingly, in order to know the exact nature of a venom, the chemical characterization of whole components may be important. In this viewpoint, we investigated the peptide component profile of the venom of *Xylocopa appendiculata circumvolans*, a solitary bee inhabiting in Japan, by peptidomic analysis using liquid chromatography-electrospray ionization-mass spectrometry (LC-ESI-MS) [5, 10]. Furthermore, we isolated two major peptides, designated xylopin and xylopinin, and found that they belong to linear cationic α-helical peptides. Biological characterization of xylopin revealed that it is an antimicrobial and cytolytic peptide.

## Methods

### LC-ESI-MS

The crude venom was analyzed with a LC (Accela 600 Pump, Thermo Scientific) connected with ESI-FTMS (LTQ Orbitrap XL, Thermo Scientific). About 10% of crude venom from a single specimen diluted in 10 μL of water was subjected to reversed-phase HPLC using CAPCELL PAK C_18_ UG 120, 1.5 × 150 mm (Shiseido Co., Ltd., Japan) with linear gradient from 5% to 65% CH_3_CN/H_2_O/0.1% formic acid at a flow rate of 200 μL/min over 20 min at 25 °C. ESI-FTMS was operated by Xcalibar software (Thermo Scientific) as: capillary voltage, + 4.6 kV; capillary temp., 350 °C; sheath and aux gas flow, 50 and 30, respectively (arbitrary units). MS/MS spectra were obtained by data dependent MS/MS mode (two most intense peaks by HCD) and the obtained spectra were manually analyzed to give peptide sequences, which were confirmed by MS-Product in ProteinProspector program (http://prospector.ucsf.edu/prospector/cgi-bin/msform.cgi?form=msproduct).

### MALDI-TOF MS

MALDI-TOF MS spectra were acquired on an Autoflex TOF/TOF mass spectrometer (Bruker Daltonics, Japan) equipped with 337 nm pulsed nitrogen laser under reflector mode. The accelerating voltage was 20 kV. Matrix, α-cyano-4-hydroxycinnamic acid (Aldrich), was prepared at a concentration of 10 mg/mL in 1:1 CH_3_CN/ 0.1%TFA. External calibration was performed with [Ile^7^]-angiotensin III (*m/z* 897.51, monoisotopic, Sigma) and human ACTH fragment 18–39 (*m/z* 2465.19, monoisotopic, Sigma). The sample solution (0.5 μL) dropped onto the MALDI sample plate was added to the matrix solution (0.5 μL) and allowed to dry at room temperature. For TOF/TOF measurement, argon was used as a collision gas and ions were accelerated at 19 kV. The series of *b* and *y* ions were afforded, which enabled identification of whole amino acid sequence by manual analysis.

### Purification

Female bees of *Xylocopa appendiculata circumvolans* were collected at Kami-ichi, Toyama in Japan. The venom sacs from five individuals were dissected immediately after collection and extracted with 1:1 acetonitrile-water containing 0.1% TFA (CH_3_CN/H_2_O/0.1% TFA), and lyophilized.

**Fig. 1:**
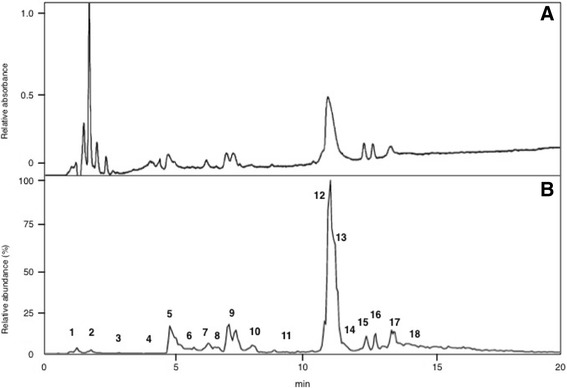
LC-ESI-MS profile of crude venom extracts of *Xylocopa appendiculata circumvolans*. About 10% of crude venom extract of a single specimen was subjected to reverse-phase HPLC using CAPCELL PAK C_18_ (1.5 × 150 mm) with linear gradient of 5–65% CH_3_CN/H_2_O/0.1% formic acid over 20 min at flow rate of 200 μL/min. **a** UV absorption by PDA. **b** Total ion current (TIC). Numbers in B show “virtual” fraction number as in Tables 2 to 6

The lyophilized extracts were subjected to reversed-phase HPLC (Shimadzu Corp., Japan) using CAPCELL PAK C_18_, 6 × 150 mm (Shiseido Co., Ltd., Japan) with a linear gradient from 5% to 65% CH_3_CN/H_2_O/0.1% TFA at a flow rate of 1 mL/min over 30 min (Fig. 1). This process released xylopin and xylopinin eluted at 25.1 min and 26.0 min, respectively.

### Amino acid sequencing

Automated Edman degradation was performed by a gas-phase protein sequencer PPSQ-10 (Shimadzu Corp., Japan).

### Peptide synthesis

Peptides were synthesized on an automated PSSM-8 peptide synthesizer (Shimadzu Corp., Japan) by stepwise solid-phase method using *N*-9-fluorenylmethoxycarbonyl (Fmoc) chemistry. All the resins and Fmoc-L-amino acids were purchased from HiPep Laboratories (Kyoto, Japan). Cleavage of the peptide from the resin was achieved by treatment with a mixture of TFA/H_2_O/triisopropylsilane (TIS) (95:2.5:2.5) at room temperature for 2 h. After removal of the resin by filtration and washing twice with TFA, the combined filtrate was added dropwise to diethyl ether at 0 °C and then centrifuged at 3000 rpm for 10 min. Thus, obtained crude synthetic peptide was purified by semipreparative reverse-phase HPLC using CAPCELL PAK C_18_, 10 × 250 mm with isocratic elution of 40–60% CH_3_CN/H_2_O/0.1% TFA at a flow rate of 3 mL/min. The homogeneity and the sequence were confirmed by MALDI-TOF MS [*m/z* 1939.1 (M + H)^+^] and analytical HPLC (co-eluted with natural peptide by using CAPCELL PAK C_18_, 6 × 150 mm with isocratic elution of 45% CH_3_CN/H_2_O/0.1% TFA at a flow rate of 1 mL/min).

### Antimicrobial activity (determination of minimal inhibitory concentration, MIC)

The microorganisms used in this study were: *Staphylococcus aureus* ATCC 25923; *Micrococcus luteus* ATCC 10240; *Bacillus subtilis* ATCC 6633; clinical isolates of: *Staphylococcus epidermidis*, *Streptococcus pyogenes, Streptococcus agalactiae, Enterococcus faecalis, Enterococcus faecium*; *Escherichia coli* ATCC 25922; clinical isolates of: *Shigella boydii, Klebsiella pneumoniae, Enterobacter cloacae, Proteus mirabilis*, *Morganella morgannii*; *Pseudomonas aeruginosa* ATCC 27853; *Stenotrophomonas maltophilia* ATCC 13637; *Acinetobacter baumanii/calcoaceticus* (clinical isolate); *Saccharomyces cerevisae* and *Candida albicans* ATCC 90112*.*

The MICs of the tested peptide were determined in the following form: 50 μL of bacterial suspension (10^6^ CFU/mL) in each well of a 96-well microtitre plates were incubated at 37 °C for 18 h with various concentration of 50 μL of the peptide solution, resulting in a final volume of 100 μL with 10^4^ CFU/well, according CLSI [11]. Following incubation, microbial growth was measured by monitoring the optical density (OD) increase at 595 nm in an ELISA reader (Multiskan® EX Thermo Fisher Scientific, EUA). The results were expressed as inhibition percentage of OD against a control (microorganisms in the absence of peptide). In addition, the lowest concentration of peptide at which there is no visible growth after overnight incubation was observed.

### Mast cell degranulating activity

The ability of the peptides to induce mast cell degranulation was investigated in vitro using the protocol of quantification of the granular enzyme β-hexosaminidase released in the supernatants of PT18 cells (a connective tissue-type mast cell model) and RBL-2H3 cells (a mucosal-type mast cell model), according to Ortega et al. [12]. For this, 4 × 10^6^ PT18 cells or 1.2 × 10^5^ RBL-2H3 cells (200 μL) were incubated in the presence of the peptides for 30 min in Tyrode’s solution at 37 °C/5% CO_2_. After this, the cells were centrifuged and the supernatants were collected. The cells incubated only with the Tyrode’s solution were lysed with 200 μL of 0.5% Triton X-100 (Sigma-Aldrich) solution to evaluate the total enzyme content. From each experimental sample to be assayed, four aliquots (10 μL) of the supernatant were taken to separate microwell plates. To these samples, 90 μL of the substrate solution containing 1.3 mg/mL of p-nitrophenyl-N-acetyl-β-D-glucosamine (Sigma Chemical Co.) in 0.1 M citrate, pH 4.5, was added and the plates incubated for 12 h at 37 °C. The reactions were stopped by addition of 100 μL of 0.2 M glycine solution, pH 10.7, and the optical density determined at 405 nm in an ELISA reader (Labsystems Multiskan Ex). The extent of secretion was expressed as the net percentage of the total β-hexosaminidase activity in the supernatant of unstimulated cells. The results represent the mean of quadruplicate tests ± standard deviation (SD).

### Hemolytic activity

The use of mice in this assay was in agreement with the Ethical Principles in Animal Research adopted by the Brazilian College of Animal Experimentation and was approved by the Ethical Committee for Animal Research of Butantan Institute (protocol no. 459/08).

To evaluate the pore-forming interaction of the peptide with biological murine membranes, a hemolytic assay was performed. A 4% suspension of mouse erythrocytes (ES) was prepared as previously described [13, 14]. Different concentrations of the peptide were incubated with the ES at room temperature (±22 °C) in an ELISA plate (96 wells) for 1 h and centrifuged (1000×*g* for 5 min). The hemolytic activity of the supernatant was measured by the absorbance at 540 nm using the absorbance of the Krebs-Henseleit physiological solution (in mM: NaCl, 113; KH_2_PO_4_, 1.2; KCl, 4; MgSO_4_, 1.2; CaCl_2_, 2.5; NaHCO_3_, 25; and glucose, 11.1), which was the vehicle for the peptide, as a blank. Total hemolysis was obtained with 1% Triton X-100, and the percentage of hemolysis was calculated relative to this value.

### Leishmanicidal activity

Medium 199 was used for the cultivation of promastigote forms of *Leishmania major* (MHOM/SU/73/5ASKH). Promastigotes were cultured in the medium [supplemented with heat-inactivated (56 °C for 30 min) fetal bovine serum (10%)] at 27 °C, in a 5% CO_2_ atmosphere in an incubator [15].

The leishmanicidal effects of the peptides were assessed using the improved 3-[4,5-dimethylthiazol-2-yl]-2,5-diphenyltetrazolium bromide (MTT assay) method as follows. Cultured promastigotes were seeded at 4 × 10^5^/50 mL of the medium per well in 96-well microplates. Then, 50 mL of different concentrations of test compounds dissolved in a mixture of DMSO and the medium were added to each well. Each concentration was tested in triplicate. The microplate was incubated at 27 °C in 5% CO_2_ for 48 h. TetraColor ONE (10 mL) a mixture of 2-(2-methoxy-4-nitrophenyl)-3-(4-nitrophenyl)-5-(2,4-disulfophenyl)-2*H*–tetrazolium,monosodium salt and 1-methoxy-5-methylphenazinium methosulfate was added to each well and the plates were incubated at 27 °C for 6 h. Optical density values (test wavelength 450 nm; reference wavelength 630 nm) were measured using a microplate reader (Thermo BioAnalysis Japan Co., Ltd.). The values of 50% inhibitory concentration of the peptides were estimated from the dose-response curve.

### Channel-like incorporation in mimetic lipid bilayers

The experiments were performed with the automated Patch-Clamp device Port-a-Patch (Nanion Technologies, Germany), using borosilicate glass chips NPC-1 with aperture diameter of approximately 10 μm. The resistance of the apertures was approximately 1 MΩ in 500 mM KCl solution. Current signals resulting from pore formation were amplified by EPC-10 amplifier (Heka Elektronik, Lambrecht, Germany) and recorded in computer after conversion performed by an analogical/digital interface ITC-1600. The system was computer controlled by the PatchControl™ software (Nanion) [16, 17].

Symmetrical solutions of 150 or 500 mM KCl with 5 mM Tris were used. Asolectin (Sigma), a negatively charged mixture of lipids, was used to form artificial membranes. Asolectin was dissolved in n-decane at a concentration of 2 mg/mL. The bilayers were painted onto the aperture of the chip using disposable polypropylene pipet tips. Measurements of the capacitive currents evoked by control voltage pulses and increase in the membrane resistance indicated the formation of bilayers. After the formation of a lipid bilayer (Rm > 1 GΩ), xylopin diluted with Milli-Q water at a 10 μM concentration was added to the *cis* side of the chip (top) to observe the single channel activity. The volume of peptide solution was never superior to 10% of the solution at the *cis* side. Voltage pulses were applied at the *trans* side of the chip (bottom). Usually, single channel activity started approximately 10 min after adding the peptide, as monitored by a constant V_hold_ of −100 mV or 100 mV. Pore conductance of incorporated channels was determined under positive and negative voltage pulses (V_hold_). The experiments were performed at room temperature (~22 °C). The data were analyzed by PatchMaster and Matlab softwares.

## Results

### On-line mass fingerprinting

LC-ESI-MS profile is shown in Fig. 1. The volume of peptide solution never exceeded 10% of the amount of crude venom from a single specimen, which is sufficient for LC-ESI-MS analysis (mass fingerprinting and peptide sequencing). On-line mass fingerprint was prepared from TIC by “virtual fractionation”, collecting MS spectra from a certain range of retention time, and then, the molecular mass was analyzed in each fraction. The results are summarized in Table 2. A total of 124 molecular mass peaks were found from 18 virtual fractions. The low molecular mass components (*m/z* 100–300) are free amino acids, biogenic amines and nucleic acids (data not shown) and those of *m/z* range from 500 to 4000 should be peptides, in particular, *m/z* from 500 to 2000 accounts for 60%, implying that a majority of components in this venom are relatively small peptides.

**Table 2:** Mass fingerprint of crude venom from *X. appendiculata circumvolans*

Fraction no.	Retention time (min)	[M + H]^+^ *m/z*
1	1.0–1.5	116.071, 175.119, 184.073, 348.071, 381.080, 405.236, 441.101
2	1.5–2.0	132.102, 268.104, 322.077, 377.058, 733.323, 759.499
3	2.0–3.0	284.099, 373.281, 437.051, 469.277, 654.357, 817.504, 947.484, 1002.508
4	3.0–4.2	182.117, 431.214, 598.428, 937.390, 1368.747
5	4.2–5.0	623.423, 646.423, 1517.714, 2064.031
6	5.0–6.0	930.588, 969.653, 1165.500, 1189.459, 1338.913, 1306.915, 1715.808, 2807.314
7	6.0–6.4	322.176, 393.214, 961.656, 1210.815, 1451.994, 1925.941
8	6.4–7.0	714.306, 1211.799, 1268.821, 1336.563, 1510.001, 3225.624, 3243.642
9	7.0–8.0	637.346, 715.291, 793.482, 838.421, 875.527, 1082.720, 1178.861, 1337.549, 1565.078, 1636.116, 2064.036, 3400.643
10	8.0–9.0	690.456, 747.477, 908.509, 933.515, 937.678, 1249.897, 1427.606, 1608.000, 1626.012, 1694.121, 2065.019, 3187.725, 3245.730
11	9.0–10.0	379.114, 506.298, 696.502, 761.492, 988.656, 1021.593, 1085.709, 1366.821, 1384.832, 1882.253, 3187.723, 3245.728
12	10.0–10.8	370.199, 941.598, 1690.115, 1735.185, 1939.274
13	10.8–11.4	619.381, 926.118, 1553.055, 1783.363, 1997.276
14	11.4–12.0	1311.877, 1803.199, 1899.267, 2087.312, 2139.353, 2236.641, 3087.760, 3256.821
15	12.0–12.7	680.314, 704.272, 1424.961, 1956.252, 2121.345, 2197.359, 2281.379, 2648.565
16	12.7–13.2	2095.280, 2113.292
17	13.2–13.6	662.303, 1316.450, 2077.267, 2153.287, 2171.297
18	13.6–15.0	1709.063, 3860.509, 4015.525

### Peptide sequencing by MS/MS analysis

Data dependent MS/MS measurement afforded MS/MS spectra from 79 peptide molecules. Manual sequence analysis of these MS/MS spectra revealed the full sequence of 58 peptides, and the rest of the 21 peptides were only partially sequenced (data not shown). The analyzed full sequences are shown in Table 3.

**Table 3:** Peptide sequences analyzed from MS/MS spectra

Fraction no.	[M + H]^+^	Sequence	Fraction no.	[M + H]^+^	Sequence
1	405.236	HLH-NH_2_	9	637.346	FAFPR
				793.482	FLVSSLK
				838.421	SNFAFPR
				875.527	GFVALLKK
				1082.720	LPLILKHLH-NH_2_
				1178.861	LLKKLPLILK
				1337.549	DGLDEYEPEDR
				1565.078	LLKKLPLILKHLH-NH_2_
				1636.116	ALLKKLPLILKHLH-NH_2_
2	759.499	ILKHLH-NH_2_	10	690.456	FVALLK
				747.477	GFVLKK
				908.509	DFLVSSLK
				937.678	LKKLPLIL
				1249.897	ALLKKLPLILK
				1694.121	ALLKKLPLILKHLHG
3	373.281	ILK	11	506.298	GFVAL
	469.277	HVLT		696.502	PLKLI
	817.504	ILKHLHG		988.656	GFVALLKKL
	947.489	EMKSVEPK		1021.593	LDFLVSSLK
				1085.709	GFVALLKKLP
				1882.253	FVALLKKLPLILKH
4	431.214	SVEP	12	1690.115	GFVALLKKLPLILKH
	598.428	LKKLP		1735.185	VALLKKLPLILKHLH-NH_2_
	937.390	EYEPEDR		1939.274	GFVALLKKLPLILKHLH-NH_2_
5	623.423	LILKH	13	619.381	GFVALL
	646.413	LVSSLK		1553.055	GFVALLKKLPLILK
				1997.276	GFVALLKKLPLILKHLKG
6	930.588	LILKHLHG	14	1311.877	GFVALLKKLPLI
	969.653	PLILKHLH-NH_2_		1803.199	GFVALLKKLPLILKHL
	1165.500	LDEYEPEDR		1899.267	GFVALLKKLPLILKHLP-NH_2_
	1338.913	KKLPLILKHLH-NH_2_		2139.353	EAGFVALLKKLPLILKHLH-NH_2_
	1396.915	KKLPLILKHLHG			
7	322.176	GFV	15	1424.961	GFVALLKKLPLIL
	393.214	GFVA			
	961.656	KLPLILKH			
	1210.815	KLPLILKHLH-NH_2_			
	1451.994	LKKLPLILKHLH-NH_2_			
8	1211.799	KLPLILIKHLH			
	1268.821	KLPLILKHLHG			
	1336.563	NGLDEYEPEDR			
	1500.001	LKKLPLILKHLHG			

These sequences can be classified according to homology and similarity. Most of them are related to the major peptide xylopin (mentioned below). As shown in Table 4, most of them are truncated peptides from both N- and C-terminus, in other words, they have a partial structure of xylopin. Seemingly, these truncated peptides are cleavage products of xylopin in some way, but it is not sure whether they are originally contained in the venom or not. Table 5 summarizes the peptides that have a similar partial sequence to xylopin as well, but no amidated C-terminus and have G (glycine) at the C-terminus instead. They are clearly the precursors of amidated C-terminus counterparts because the C-terminal amidation (post-translational modification) takes place by oxidation-hydrolysis of C-terminal G (glycine) residue.

**Table 4:** Peptides related to xylopin

Fraction no.	[M + H]^+^	Sequence
7	322.176	GFV
7	393.214	GFVA
11	506.502	GFVAL
13	619.381	GFVALL
10	747.477	GFVALLK
9	875.527	GFVALLKK
11	988.656	GFVALLKKL
11	1085.709	GFVALLKKLP
14	1311.877	GFVALLKKLPLI
15	1424.961	GFVALLKKLPLIL
13	1553.055	GFVALLKKLPLILK
12	1690.115	GFVALLKKLPLILKH
14	1803.199	GFVALLKKLPLILKHL
3	373.281	ILK
5	623.423	LILKH
7	961.656	KLPLILKH
8	1211.799	KLPLILKHLH
9	1178.861	LLKKLPLILK
10	690.456	FVALLK
10	1249.897	ALLKKLPLILK
10	937.678	LKKLPLILK
11	696.502	LPLILK
1	405.236	HLH-NH_2_
2	759.499	ILKHLH-NH_2_
6	969.653	PLILKHLH-NH_2_
9	1082.720	LPLILKHLH-NH_2_
7	1210.815	KLPLILKHLH-NH_2_
6	1338.913	KKLPLILKHLH-NH_2_
7	1451.994	LKKLPLILKHLH-NH_2_
9	1565.078	LLKKLPLILKHLH-NH_2_
9	1636.116	ALLKKLPLILKHLH-NH_2_
12	1735.185	VALLKKLPLILKHLH-NH_2_
11	1882.253	FVALLKKLPLILKHLH-NH_2_
14	1899.267	GFVALLKKLPLILKHLP-NH_2_^a^
12	1939.274	GFVALLKKLPLILKHLH-NH_2_^b^
19	2139.353	EAGFVALLKKLPLILKHLH-NH_2_

^a^Xylopinin, ^b^xylopin

**Table 5:** Peptides without amidated C-terminus

Fraction no.	[M + H]^**+**^	Sequence
3	817.504	ILKHLHG
6	930.588	LILKHLHG
8	1268.821	KLPLILKHLHG
6	1396.915	KKLPLILKHLHG
8	1510.001	LKKLPLILKHLHG
10	1694.121	ALLKKLPLILKHLHG
13	1997.276	GFVALLKKLPLILKHLHG

The rest of the peptides in this venom may be new peptides as summarized in Table 6. All these have no homology to any known peptides.

**Table 6:** Unknown peptides

Fraction no.	[M + H]^+^	Sequence
3	469.277	HVLT
3	654.357	EVLSAH-NH_2_
4	431.214	SVEP
3	947.484	EMKSVEPK
9	637.346	FAFPR
9	838.421	SNFAFPR
5	646.413	LVSSLK
9	793.482	FLVSSLK
10	908.509	DFLVSSLK
11	1021.593	LDFLVSSLK
4	937.390	EYEPEDR
6	1165.500	LDEYEPEDR
8	1336.563	NGLDEYEPEDR
9	1337.549	DGLDEYEPEDR

### Purification and sequence determination of major peptides

**Fig. 2:**
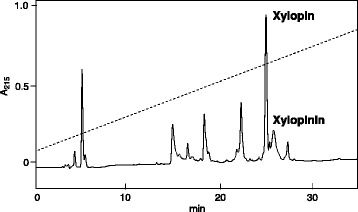
Fractionation of venom extracts of *Xylocopa appendiculata circumvolans* by reverse-phase HPLC using CAPCELL PAK C_18_ (6 × 150 mm) with linear gradient of 5–65% CH_3_CN/H_2_O/0.1% TFA over 30 min at flow rate of 1 mL/min. UV absorption was monitored at 215 nm

Two major peptides, called xylopin and xylopinin, were purified by reversed-phase HPLC (Fig. 2). The primary sequence of xylopin was determined by Edman degradation as GFVALLKKLPLILKHLH, which corresponded to a peptide component with *m/z* 1939.274 (M + H)^+^ in the crude venom, and accordingly, the C-terminus is amidated. The solid-phase synthesis of this peptide and the HPLC comparison of the synthetic specimen with the natural peptide finally corroborated the sequence.

The sequence of xylopinin was determined by MALDI-TOF/TOF analysis as GFVALLKKLPLILKHLP-NH_2_, in which L and I were differentiated by *w* and *d* ions, and corresponded to the peptide with *m/z* 1899.267 (M + H)^+^ in the crude venom.

**Fig. 3:**
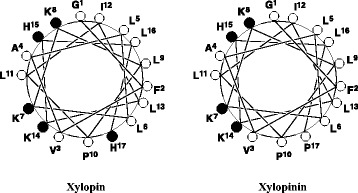
Helical wheel projection of the sequence of xylopin and xylopinin. In this view through the helix axis, the hydrophilic His (H) and Lys (K) residues are located on one side and the hydrophobic Ala (A), Phe (F), Ile (I) and Leu (L) residues on the other side of the helix

The chemical features of xylopin and xylopinin, rich in hydrophobic and basic amino acids with no disulfide bond, are characteristic of linear cationic cytolytic peptides [18]. The known solitary bee venom peptides, melectin and osmin, can be included in this type of peptides, and are highly homologous to these new peptides. This class of peptides has been known to adopt an amphipathic α-helical conformation, showing an amphiphilic character under appropriate conditions [19–22], and the amphipaticity of peptides has been considered essential for their biological activities [23]. In fact, if the helical wheel projection of xylopin and xylopinin sequences were drawn, amphipathic α-helical conformations would be depicted as in Fig. 3. Based on this view, all the hydrophilic amino acid residues, S, H and K, are located on one side, whereas the hydrophobic amino acid residues, A, F, I, L and V are on the other side of the helix.

### Biological activities

Biological activities of xylopin were evaluated by using synthetic specimen. The mast cell degranulation, hemolysis, antimicrobial and antiprotozoan (leishmanicidal) activities were tested because these are characteristic biological activities for these types of peptide.

**Fig. 4:**
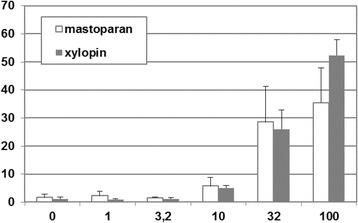
The degranulation in RBL-2H3 cells (a mucosal-type mast cell model) measured by the β-hexosaminidase release, basal and after treatment with xylopin, the novel venom peptide from the solitary bee *Xylocopa appendiculata circumvolans*. Concentrations are in μM and data represent the mean from two to four independent experiments

Mast cell degranulation activity on RBL-2H3 cells was similar to mastoparan at low concentrations (<30 μM), whereas at higher concentrations (100 μM), it was more potent than mastoparan (Fig. 4). Antimicrobial activity can be considered strong and of broad spectrum, with MICs from 1.9 to 15 μM. The peptide showed the lowest MIC values against gram-positive bacteria, with exception of *S. aureus* ATCC25923 and *Enterococcus* spp., and presented potent activities against yeasts (Table 7). Hemolytic activity against mouse erythrocytes, however, was low, reaching only 30% at the highest concentration of 1 mM. Xylopin showed significant leishmanicidal activity with an IC_50_ of 25 μM against *Leishmania major*.

**Table 7:** Minimum inhibitory concentration (MIC) of xylopin

Microorganism	MIC (μM)
Gram-positive	
* Staphylococcus aureus* ATCC 25923	15.0
* Micrococcus luteus* ATCC 10240	1.9
* Bacillus subtilis* ATCC 6633	3.75
* Streptococcus pyogenes* (CS)	3.75
* Streptococcus agalactiae* (CS)	3.75
* Enterococcus. faecalis* (CS)	15.0
Gram-negative	
* Escherichia coli* ATCC 25922	3.75
* Pseudomonas aeruginosa* ATCC 27853	7.5
* Stenotrophomonas maltophilia* ATCC 13637	3.75
* Shigella boydii* (CS)	3.75
* Klebsiella pneumoniae* (CS)	7.5
* Serratia marcescens* (CS)	7.5
* Enterobacter cloacae* (CS)	7.5
* Proteus mirabilis* (CS)	>30
* Morganella morgannii* (CS)	30
* Acinetobacter baumanii/calcoaceticus* (CS)	3.75
Yeast	
* Candida albicans* ATCC 90112	7.5
* Sacharomyces cerevisae*	3.75

### Channel-like incorporation in mimetic lipid bilayers

**Fig. 5:**
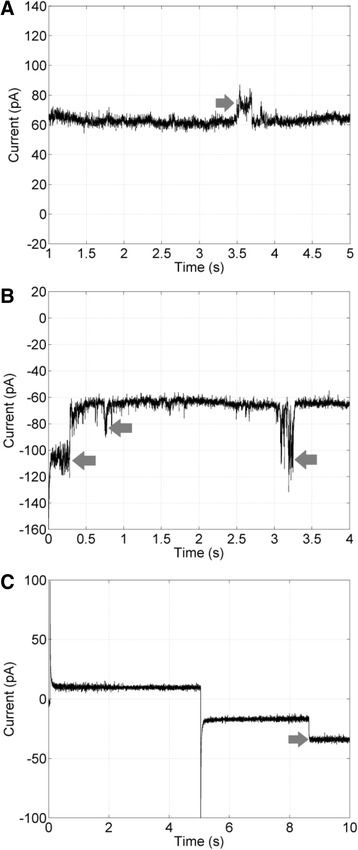
Representative recordings of single channel incorporation in asolectin artificial lipid bilayers induced by xylopin at 1 μM concentration. **a** Vhold = + 140 mV, pore conductances = 63 and 105 pS. **b** Vhold = −140 mV, pore conductance = 143 and 259 pS. **c** Vhold was set at +100 mV for 5 s and was switched to −100 mV for the remaining 5 s, pore conductance = 175 pS. Solutions: **a** and **b** 150 mM KCl, and **c** 500 mM KCl (symmetrical). Arrows indicate channel apertures or closings. Four independent experiments were performed

Xylopin induced pore formation in painted asolectin artificial lipid bilayers at 1 μM concentration. The apertures occurred when voltage was clamped at either positive or negative values. Pores of different conductance levels (from 45 to 260 pS in a 150 mM KCl solution, V_hold_ ± 140 mV; and from ~75 to 175 pS at V_hold_ ± 100 mV in a 500 mM KCl solution) were recorded in our experiments (Fig. 5).

## Discussion

In this study, we have analyzed all the components in the crude venom of *Xylocopa appendiculata circumvolans*, a solitary bee inhabiting from Japan, by using LC-ESI-MS and MS/MS. It revealed that this venom contained 124 components and most of them are small peptides. The peptide sequences were further analyzed by manual analysis of their MS/MS spectra, which led to the determination of whole sequence of 58 peptides. However, most of them are related to the major peptide xylopin, having a truncated partial sequence of xylopin. Therefore, these peptides may come from cleavage of xylopin in some way, but it is not clear whether they are originally contained in the venom or not.

Most notably, these results were obtained by using only 10% of the amount of a single venom content. Among the hymenopteran insect venoms, solitary bee venom has not been extensively studied yet. One of the reasons for this may come from the difficulty of collecting a sufficient amount of venom for chemical analysis, which is due to the insect solitary life style. However, as shown in this study, the remarkable progress of mass spectrometry in sensitivity made it possible to perform this type of peptidomic analysis with so minute amount of venom. It is particularly advantageous not only for solitary bee venom but also for solitary wasp venom, and the studies along this line are in progress in our laboratory.

In addition to peptidomic analysis, we have purified and characterized the major peptide components, xylopin and xylopinin, by the conventional method. The chemical and biological characteristics of xylopin are similar to the known solitary bee venom peptides – melectin and osmin – and, accordingly, this novel peptide belongs to the linear cationic α-helical peptide group. Xylopin presented broad-spectrum antimicrobial activity, with very low hemolytic activity. Xylopin has also a Pro10 residue in the sequence, in a similar position as both melectin and osmin that present a Pro11. According to Cerovsky et al. [4], the Pro11 residue conferred to this peptide selectivity to the antimicrobial activity, as well as low hemolytic activity.

The pore formation by xylopin in artificial lipid bilayers was confirmed through electrical measurements. This is the first report of solitary bee venom peptides inducing pore formation in artificial lipid bilayers. Asolectin was employed because it is negatively charged and has shown to be a good membrane model for this class of peptide in previous studies [14, 24]. Conductance of the pores formed by xylopin was bigger than the conductance of the pores formed by the eumenitin-R and F, and EMP-ER and -EF from solitary wasp venoms [14]. Additionally, large conductance pores (>500 pS) were not observed in the presence of xylopin, similarly with the eumenine mastoparan peptides EMP-ER and -EF [14], probably due to their amidated C-terminal, that would prevent cluster formation constituted by several units of the peptide.

## Conclusions

LC-ESI-MS and MS/MS analysis of the crude venom extract from a solitary bee *Xylocopa appendiculata circumvolans* revealed the component profile of this venom, which mostly consisted of small peptides. The major peptide components, xylopin and xylopinin, were purified and characterized by the conventional technique. Their chemical and biological characteristics, belonging to linear cationic α-helical peptides, are similar to the known solitary bee venom peptides, melectin and osmin. Pore formation in artificial lipid bilayers was demonstrated for the first time with a solitary bee peptide.

## Abbreviations

CS: Clinical sample
ES: Erythrocytes
ESI-FTMS: Electrospray ionization-Fourier transformed mass spectrometry
FTMS: Fourier transformed mass spectrometry
HPLC: High performance liquid chromatography
LC: Liquid chromatographty
LC-ESI-MS: Liquid chromatography-elctrospray ionization-mass spectrometry
MALDI-TOF MS: Matrix assisted laser desorption/ionization time-of-flight mass spectrometry
MIC: Minimal inhibitory concentration
MS: Mass spectrometry
MS/MS: Tandem mass spectrometry
OD: Optical density
SD: Standard deviation
TIC: Total ion current
TOF/TOF: Time-of-flight/time-of-flight
